# Individual differences in rate of acquiring stable neural representations of tasks in fMRI

**DOI:** 10.1371/journal.pone.0207352

**Published:** 2018-11-26

**Authors:** Ming-Hua Chung, Bradford Martins, Anthony Privratsky, G. Andrew James, Clint D. Kilts, Keith A. Bush

**Affiliations:** Brain Imaging Research Center, Psychiatric Research Institute, Department of Psychiatry, College of Medicine, University of Arkansas for Medical Sciences, Little Rock, Arkansas, United States of America; Centro de Neurociencias de Cuba, CUBA

## Abstract

Task-related functional magnetic resonance imaging (fMRI) is a widely-used tool for studying the neural processing correlates of human behavior in both healthy and clinical populations. There is growing interest in mapping individual differences in fMRI task behavior and neural responses. By utilizing neuroadaptive task designs accounting for such individual differences, task durations can be personalized to potentially optimize neuroimaging study outcomes (e.g., classification of task-related brain states). To test this hypothesis, we first retrospectively tracked the volume-by-volume changes of beta weights generated from general linear models (GLM) for 67 adult subjects performing a stop-signal task (SST). We then modeled the convergence of the volume-by-volume changes of beta weights according to their exponential decay (ED) in units of half-life. Our results showed significant differences in beta weight convergence estimates of optimal stopping times (OSTs) between go following successful stop trials and failed stop trials for both cocaine dependent (CD) and control group (Con), and between go following successful stop trials and go following failed stop trials for Con group. Further, we implemented support vector machine (SVM) classification for 67 CD/Con labeled subjects and compared the classification accuracies of fMRI-based features derived from (1) the full fMRI task versus (2) the fMRI task truncated to multiples of the unit of half-life. Among the computed binary classification accuracies, two types of task durations based on 2 half-lives significantly outperformed the accuracies using fully acquired trials, supporting this length as the OST for the SST. In conclusion, we demonstrate the potential of a neuroadaptive task design that can be widely applied to personalizing other task-based fMRI experiments in either dynamic real-time fMRI applications or within fMRI preprocessing pipelines.

## Introduction

Task-based functional magnetic resonance imaging (fMRI) is a now widely-used methodology for studying the neural information processing states related to engaged human behaviors for both healthy and clinical populations. For the great majority of task-based fMRI approaches, the task parameters (quantity, trial types, and temporal sequence of trial types) are estimated according to behavioral performance criteria that have been identified in separate (i.e., initial) samples of participants. These estimates are then generalized (often via group-level comparisons) across both study participants as well as all subsequent studies, resulting in methodological conventions, e.g., parameter choices, that do not have empirical support commensurate with their widespread adoption.

There has also been growing interest in mapping individual differences in fMRI task behavior and functional brain responses, particularly to address the barriers posed by group-level brain-behavior mapping [1]. Recent fMRI studies have explored individual differences in neural population activation on a trial-by-trial basis–specifically, by exploiting the temporal development of neural activations associated with task processing demands in order to individually titrate task parameters (such as task type, task duration, or task stimulus type) [2,3].

fMRI neuroactivation studies may capitalize on individual differences in trial-by-trial neural response variation in several ways. First, the number of discrete task trials administered to a participant (i.e. paradigm duration) may be dynamically adjusted to optimally characterize the individual’s behavioral-neural response coupling, e.g., sampling until estimation of task-evoked neural activity reaches a steady state; an approach which strengthens functional neuroanatomical inferences while simultaneously minimizing participant scanner time, discomfort, and fatigue (all of which are confounds to task performance) [3]. Second, trial types may be dynamically selected based on a pre-defined neural response criterion, e.g., selecting the next trial’s parameters to maximize the novelty of its observed neural response [4], which may individually optimize the quality of derived brain-behavior relationships or behavioral performance parameters while simultaneously minimizing scan duration. However, this latter approach is limited by the need for a pre-defined model of the stimulus-neural response relationship.

Real-time fMRI (rt-fMRI) technology is particularly well-suited to exploit task designs that explore and accommodate individual differences in task-evoked neural activity. Indeed, recent rt-fMRI applications exemplify best practices approaches to leveraging the trial-by-trial response variations, outlined above. Dosenbach et al. [5] recently applied rt-fMRI-derived motion analysis to dynamically alter the duration of task-based fMRI acquisitions to maximize task inference and reduce experiment cost. The OpenNFT toolbox [6] incorporates the ability to conduct iterative, volume-by-volume general linear model (GLM) regression to characterize the temporal dynamics of task-based neural activity in anatomical regions of interest.

For this work, we sought to exploit individual temporal variance in trial-related neural responses to estimate personalized optimal stopping times (OSTs) that reflect the number of iterated trials needed to identify a stable neural response for each trial type. This individual differences research was pursued as a basis for guiding future rt-fMRI applications to prospectively characterize and control for individual differences in the rate at which neural responses track to repeated task trial type presentations–and thus define an individually standardized neural response. For this study, OST was estimated via retrospective analysis of pre-specified fMRI tasks as a proof-of-concept for future rt-fMRI applications.

**Fig 1:**
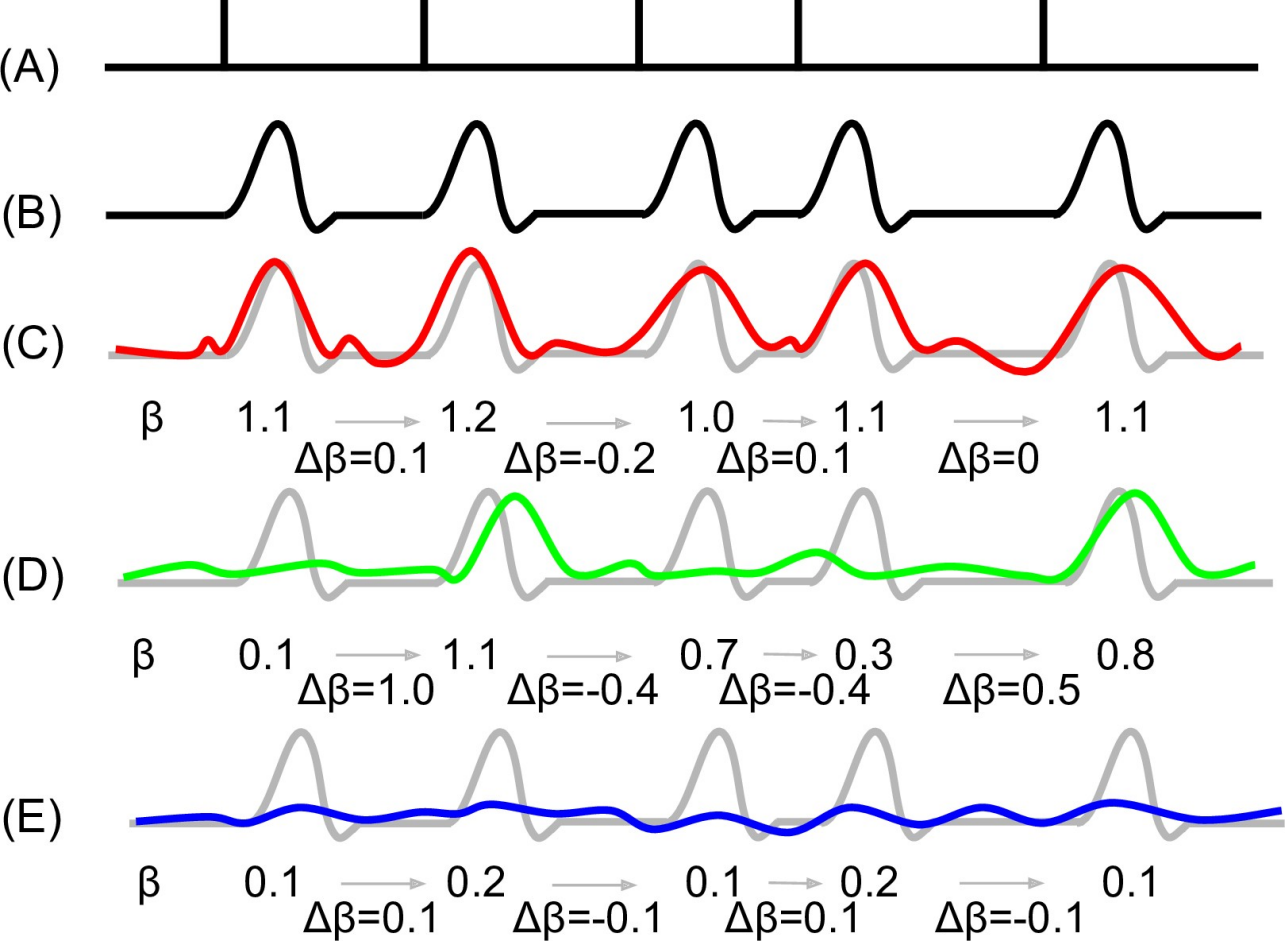
A conceptual model for exploitation of individual differences in trial-by-trial neural response variation via GLM. (A) Trial timings. Each vertical line demarks the onset of a trial. For the purpose of this conceptual model, we assume that all trials are represented by a single trial type. (B) Theoretically predicted hemodynamic response to trials presented as in panel A. (C) An example of a neural activation pattern (red) exhibiting strong response to the task and low trial-by-trial variance; hence, GLM fit represented by beta weight (β) converges rapidly. (D) An example of a neural activation response pattern (green) exhibiting mixed response to the task and high trial-by-trial variance; hence, β converges slowly. (E) An example of a neural activation pattern (blue) exhibiting weak response to the task; therefore, trial-by-trial variation is low and β converges rapidly. Note, in panels C-E the theoretically predicted hemodynamic response is provided as an underlay for visual comparison of trial-by-trial neural activation response patterns. For this study of these individually varying convergence patterns, we modeled the change of beta weights (Δβ) to estimate the point of convergence of neural activation responses.

The conceptual basis of our OST estimation model would extend the commonly used general linear model (GLM) for estimation of task-related neural activations (i.e., beta weights) to the case of volume-wise (i.e., volume-by-volume) GLM. Volume-wise GLM generates trajectories of neural activations that converge as the task-neural response relationship is sufficiently sampled (Fig 1). We hypothesized that neural activation convergence (i.e., the change of beta weights) is well-modeled by the exponential decay function, facilitating the parameterization of individual convergence rates according to a standardized unit, half-life. These derived parameters were then generalized to trial type and group-level empirical comparisons as a proof-of-concept.

Our goal here was to explore the utility of estimating individualized OSTs, measured in half-lives of beta-weight convergence, without compromising the accurate characterization of brain-behavior relationships by the use of abbreviated image acquisition. To test our OST estimation model, we first sought to characterize the degree of inter-individual variation in the convergence rate of patterns of neural activation responses for each task trial type. Second, we sought to validate the information quality contained within the individually estimated neural activation responses for discrete temporal units of convergence (i.e., half-life, *t*_1/2_) in comparison to neural responses that are typically estimated across individuals using the fully acquired canonical Stop Signal fMRI task (i.e., full task duration). To validate the information content of the *t*_1/2_-estimated neural activations, we attempted a replication of our past work using neural pattern classification to discriminate between healthy control individuals and individuals with DSM-IV cocaine dependence (CD) [7]. Specifically, we tested if *t*_1/2_-derived neural activation patterns significantly differed in binary classification accuracy from the canonical task-derived neural activation patterns. Based on inter-individual estimations of convergence speed as well as group-level classification results, we identified 2 * *t*_1/2_ to be the OST for the SST. We also tested if individual SST-related response inhibition behavior was similar between abbreviated and canonical task durations.

## Materials and methods

### Dataset

We conducted a post-hoc analysis of 134 functional MRI (fMRI) scanning sessions acquired for female and male study volunteers (18–60 years old) who underwent the Stop Signal fMRI task at the University of Arkansas for Medical Sciences (UAMS) Brain Imaging Research Center (BIRC) or the Emory University Biomedical Imaging Technology Center (BITC) from 2007–2016. All studies were conducted in accordance with the Declaration of Helsinki and with oversight and approval by the UAMS or Emory Institutional Review Boards. All data were collected with oversight and approval of the UAMS Institutional Review Board (IRB), in accordance with principles expressed in the Declaration of Helsinki. Subjects provided written informed consent to participate under the following UAMS IRB protocols: #113160, 137634, and 202715. Specifically, the informed consent process included participants’ permission to collect and analyze behavioral and neuroimaging data. Dr. Kilts served as principal investigator for all protocols. All data have been de-identified prior to dissemination to ensure provisions of privacy and confidentiality set forth in the above protocols.

Of these 134 participants, 11 were excluded from fMRI data analysis due to artifacts arising from deep sulci or large venous structures, and 6 additional participants were excluded due to excessive head motion. One additional participant was removed due to the failure of the GLM regression to converge for the motor regressor (see volume-wise general linear regression). Because the ventral medial prefrontal cortex (vmPFC) was predicted to play a key role in error processing trials (failed Stop trials), a quality control check was performed to exclude participants who had excessive signal dropout in the vmPFC due to sinus cavity artifact. 21 participants were excluded from analyses due to vmPFC signal dropout. Of the remaining 95 participants two subgroups were assembled based upon past and present substance use and mental health history as determined by the Structured Clinical Interview for DSM IV (SCID-IV) to represent healthy controls (n = 27, 14 males, without past or present drug use and psychopathology) and CD (n = 40, 30 males, all meeting DSM-IV criteria for cocaine dependence). Classification analyses focused on discriminating the healthy control and CD subgroups (total n = 67).

### Stop signal task

The Stop Signal fMRI task measured five processes of motor behavior and response inhibition based on the component trial types (Go and Stop trials) and the success/failure of stop responses (successful trials, error trials, and post-error adaptation). The behavioral and neural correlates of this task have been intensely studied as correlates for clinical disorders of behavioral dysregulation (e.g. addiction, ADHD). The Stop Signal task is typically administered as a lengthy paradigm (typically 15–25 minutes) with prepotent Go responses; this task duration is motivated by the need to ensure sufficient sampling of trials associated with each task condition to facilitate the identification of component processes and their neural processing correlates during post-hoc modeling. In its canonical forms, the Stop Signal fMRI task weights the inclusion of sufficient modeled trials for each type for all individuals over the possible cost related to individual differences in trial-by-trial variation in the rate of acquiring stable neural responses.

Subjects underwent fMRI while performing a performance-adjusted Stop Signal task in which they were presented with a series of random alphabetical letters (“Go signal”), and instructed to press a single button with the index finger of their dominant hand as quickly as possible following stimulus presentation. They were further told to inhibit the Go stimulus response whenever a white square appeared around the letter (“Stop signal”). The Stop signal followed the Go stimulus by a short delay for 75 of the 300 trials. The inter-trial interval was fixed at 2,000 ms with the Stop signal delay (SSD) initially set to 250 ms following stimulus presentation. The SSD increased by 50 ms following successful inhibition on a Stop trial and decreased by 50 ms following an error of commission on a Stop trial. This adjustment of SSD was designed to maintain a successful stopping rate of approximately 50%. Three 20 s rest periods were presented over the course of the 16.6 min task.

### Image acquisition

MRI data were acquired using one of three instrument configurations. Among 67 used subjects, 28 subjects were acquired using a Siemens 3T TIM Trio (Siemens Healthcare, Munich, Germany) with a 12-channel head-coil. 39 subjects were acquired using a Philips 3T Achieva X-series MRI scanner (Philips Healthcare, Eindhoven, The Netherlands). Of the subjects scanned on the Philips instrument 36 subjects were acquired using an 8-channel head coil and 3 subjects were acquired using a 32-channel head coil. Anatomic images were acquired with a MPRAGE sequence (matrix = 256 x 256, 220 sagittal slices, volume/TE/FA = shortest/shortest/8°, final resolution = 0.94 x 0.94 x 1 mm^3^. Functional images were acquired with the following EPI sequence parameters: volume/TE/FA = 2000 ms/30 ms/90°, FOV = 240 x 240 mm, matrix = 80 x 80 (64 x 64 on the Siemens scanner), 37 oblique slices (32 oblique slices on the Siemens scanner), ascending sequential slice acquisition, slice thickness = 2.5 mm with 0.5 mm gap, final resolution 3.0 x 3.0 x 3.0 mm^3^.

### Image preprocessing

All MRI data preprocessing was conducted in AFNI (Version AFNI_16.3.20) [8] unless otherwise noted. Anatomic data underwent skull stripping, spatial normalization to the icbm452 brain atlas, and segmentation into white matter (WM), gray matter (GM), and cerebrospinal fluid (CSF) with FSL [9]. Functional images underwent despiking; slice correction; deobliquing (to 3x3x3 mm^3^ voxels); head motion correction including volume censoring of framewise displacements > 0.5 [10,11]; transformation to the spatially normalized anatomic image; regression of 24 motion parameters (raw, raw^2^, differential, differential^2^)[10], and regression of the mean and differential time courses for white matter voxels and CSF space voxels [10,11]; spatial smoothing to approximate an 8-mm FWHM Gaussian kernel (via 3dBlurToFWHM); temporal highpass filtering of 0.0078 Hz (i.e. 1/128 s); and, scaling to percent signal change.

### Independent component analysis

After preprocessing, functional neuroimaging datasets were reduced to spatially independent group-level networks of activation using independent component analysis (ICA) within the GIFT Matlab toolbox [12]. The use of ICA is to ensure the computational cost/time of the whole process is manageable. Notice the implementation of ICA here is not feasible in a real-time scenario. However, there are public available group-level ICA maps (e.g., Human Connectome Project [13]) if it is implemented in real-time. While these ICAs are based upon resting state data, it has been shown in earlier work that the ICA components of resting state contain the diverse set of networks observed in task activated data [14]. We see this work as a proof-of-concept retrospective study of how pre-defined ICAs may perform in a real-time scenario. Moreover, in a real-time scenario, modeling beta weights via GLM voxel-wise is potentially too slow. Therefore, compression the input dimension through ICA is essential to be able to monitor the beta weights in real-time.

Here, we utilized ICASSO [15] to estimate independent component (IC) reliability, removing components with a low stability index, I_q_<0.9, sampled from 20 runs, randomly initialized. GIFT was configured to compute PCA using expectation maximization, compressing to 60 principal components in the 1st step, and 30 principal components in the 2nd step as well as to use infomax to solve for ICAs and scale the resultant ICAs to z-scores. The size of our component set, N = 30, is comparable to the number of ICs typically found to be stable in support of machine learning-based classification. Moreover, after removal of low stability components and unusable components (ventricles and CSF), the resulting number of usable ICs (N = 22) fell within the ideal range [16]. Descriptions of the major nodes of functional networks represented by the identified ICA components are summarized in Table 1. A mean spatial map was derived from group ICA and activity timecourses for each participant and IC by projecting participants’ fMRI data into ICA-space. These ICA timecourses were used as response variables for the step-wise general linear regression described below.

**Table 1:** The full list of identified ICs.

1	CSF	16
2	CSF	17
3	Motor	18
4	Superior Medial Parietal	19
5	Bilateral Temporal Pole	20
6	VMPFC	21
7	MPFC	22
8	Uninterpretable	23
9	Left Parietal	24
10	Left Frontoparietal	25
11	Right Parietal	26
12	CSF	27
13	V1	28
14	Right Frontoparietal	29
15	V2	30

Note that there were 7 ICs related to CSF volumes and 1 uninterpretable IC, which were subsequently excluded from analysis.

### Volume-wise general linear regression

To approximate outcomes of general linear regression analysis performed at each volume in real time as a means of simulating trial-by-trial analyses, we implemented a volume-wise general linear regression (vwGLR) with a gradually increasing range of acquisition volumes. Because it is implausible that the changes in beta weights would cease to be affected by upcoming trials at the very beginning of the task, we did not start our vwGLR at the first volume. Rather, we arbitrarily selected a 49-volume “burn-in” period and initiated our vwGLR from the 50^th^ volume onward (or in a small number of cases, from the first volume for which all sub-task regressors induced non-empty regression coefficients). The response variables in our vwGLR were the ICA timecourses, described previously. The regressors in our vwGLR included 24 motion parameters, all motor responses, and 6 different trial types: instruction, Go following Go (GG), Go following Successful Stop (GfSS), Go following Failed Stop (GfFS), Successful Stop (SS), and Failed Stop (FS). We censored regression volumes according to framewise displacement (see Image preprocessing). The actual regression was performed in AFNI [8] via the 3dDeconvolve and 3dREMLfit functions. In an iterative process, starting at t = 50^th^ volume, the beta weights generated from 3dREMLfit were saved (β_t_) and vwGLR was repeated using the first t+1 volumes of the fMRI data. This process was repeated t = 50, …, T_j_ where T_j_ is the last acquired volume for the j^th^ subject. Note, the set of saved beta weights were not identical because not all subjects started vwGLR at the 50^th^ volume.

### Modeling the exponential decay of the changes in beta weights across time

**Fig 2:**
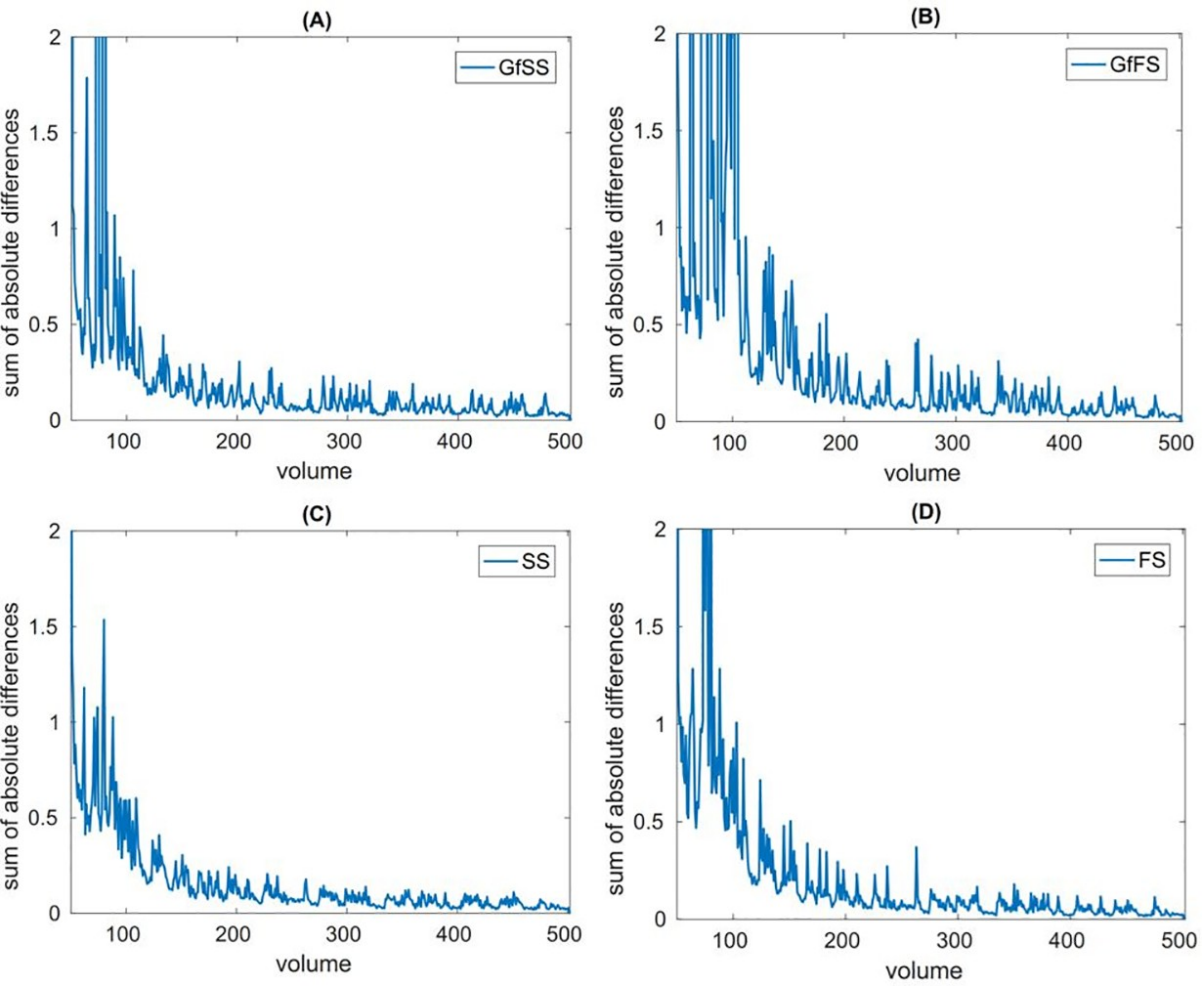
The average sum of absolute differences of beta weights (over all valid ICAs) between volume t-1 and volume t for GfSS, GfFS, SS, and FS trials. This figure shows the average SoAD values across volumes for (A) GfSS, (B) GfFS, (C) SS, and (D) FS. All 4 trial types exhibited a temporally decreasing trend consistent with the gradual stabilization of the beta weights across trials.

GLM-based studies generally assume that steady-state neural activation can be modeled for each subject and trial type, given a sufficiently long task. Consequently, this assumption underlies the temporal convergence of beta weights derived from our vwGLR; that is, we should expect the change in derived beta weights to diminish with increasing number of consecutive trials. To monitor the ongoing changes in beta weights, we calculated the sum of absolute differences (SoAD) of beta weights between volume t-1 and volume t (i.e., ∑_*t*_|*β*_*t*_−*β*_*t*−1_|) for each participant and trial type. Here, we challenged the assumption that all trial types converge simultaneously. Instead, we hypothesized that each trial type has its own convergence rate. Specifically, we focused on GfSS, GfFS, SS, and FS trial types to test this hypothesis. As shown in Fig 2, the average SoAD values among participants progressively declined across volumes for all 4 trial types of interest, indicating the gradual convergence of the beta weights.

As one of the more common functions to model signal decay, the exponential decay function has been used previously in modeling fMRI signal decay [17]. Here, we used a single exponential decay function to model the SoAD values for each participant and trial type; that is,

$$N(t)=N_{0}e^{-\lambda t},$$ (1)

where *N*(*t*) is the SoAD of beta weights at volume *t*, *N*_0_ is the intercept of the decay function (i.e., *N*_0_ = *N*(0)), and *λ* is the exponential decay constant. Natural logarithmic transformation of the equation yielded

$$\ln\left(N(t)\right)=\ln\left(N_{0}\right)+(-\lambda )t.$$ (2)

**Fig 3:**
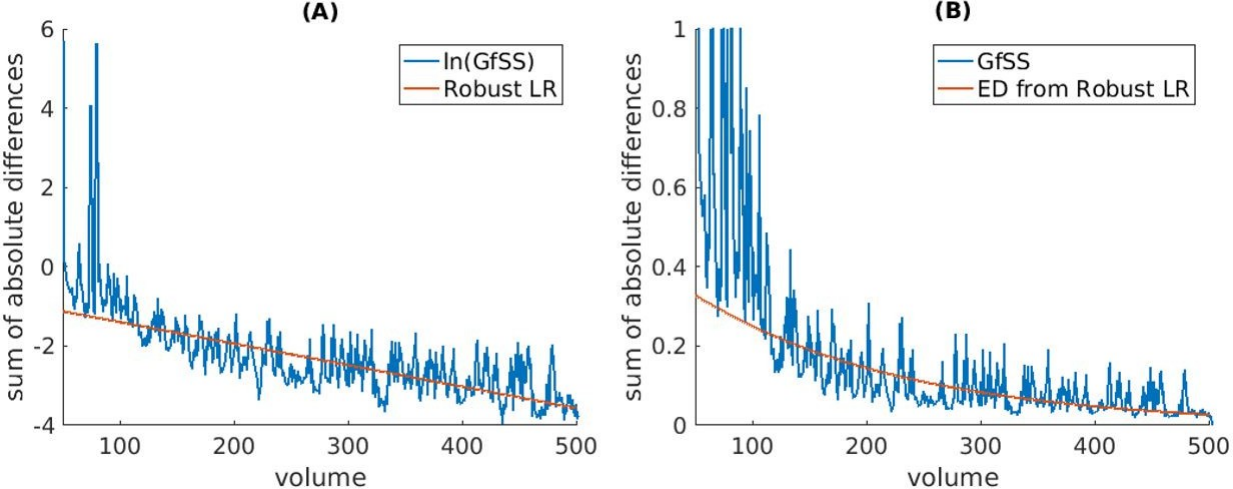
Robust linear regression on the average SoAD value timecourse for GfSS. (A) Fitting robust linear regression (Robust LR) to on the natural logarithm of the average SoAD values of GfSS betas (B) Once the decay constant *λ* is obtained by regression, we fit the SoAD values with exponential decay functions generated by its associated *λ*.

Notice that Eq 2 is mathematically equivalent to a linear function. Thus, the exponential decay constant *λ* and intercept *N*_0_ can be calculated by solving the slope (−*λ*) and intercept *ln*(*N*_0_) of Eq (2) via regression. To illustrate, we fit robust linear regression lines (via Matlab, default parameters)[18] for the natural logarithm of the average SoAD values of GfSS betas (Fig 3A). Once we calculated *λ*, we generated the associated exponential decay lines to fit the SoAD values (Fig 3B). Note that robust linear regression has the advantage of countering extreme outliers and was used in all subsequent analyses. A comparison of outcomes from ordinary and robust regression can be seen in S1 Fig.

Estimation of task convergence based on the half-life of exponential decay of SoAD values. With the exponential decay of SoAD values there is an intuitive quantity which can be used as a unit of convergence rate—half-life, which represents the time for which the exponential decay of SoAD values falls by one-half (i.e., 50%) of their original value and can be calculated directly by

$$t_{1/2}=\frac{\ln\left(2\right)}{\lambda },$$ (3)

where *t*_1/2_ denotes the half-life and λ is the decay constant derived from robust LR.

By modeling outcomes related to the elapsing of multiple half-lives (i.e., *k* * *t*_1/2_, where *k* = 1, 2, 3, 4), we can determine the estimated time that the exponential decay function for SoAD value falls 50%, 75%, 87.5%, and 93.75% (i.e., 1, 2, 3, and 4 half-lives) from its original value. In terms of our measure of interest, SoAD, the half-life estimates identify the volumes for which trial-by-trial differences in the trial type-related neural response has decreased by these quantized percentages, allowing us to identify those volumes associated with the balance of the task’s duration and the temporal convergence of beta weights to stable estimates. We computed subject-wise beta weights at trial presentation stopping times measured in units of 1, 2, 3, and 4 half-lives for each of the four SST trial types that represent each of their optimal balance states and thus optimal stopping time (OST). The OST (measured in volumes) across all four trial types was then fixed as the largest OST value for the set.

### Primary validation of *t*_1/2_-derived OSTs: estimated SST-related neural activation pattern as a clinical label

To validate the information content of the OST-related neural activation states, we tested their relative ability (in comparison to activation patterns derived from the fully acquired data set) to discriminate healthy control individuals from individuals with DSM-IV cocaine dependence (CD) [7]. Among the 67 usable subjects in our study, we used the cocaine-dependent (CD) subsample (n = 40) and the control (CON) subsample (n = 27) to train and test a support vector machine (SVM) classifier [19], implemented via Matlab’s fitcsvm function [18] with linear kernel and default cost settings.

Classification features for each of the four modeled stopping times (1, 2, 3, and 4 half-lives) were formed by concatenation of all the vwGLR-derived beta weights (GfSS, GfFS, SS, and FS trials). If any OST estimate exceeded a subject’s last (fully acquired) volume, then the beta weights of the last volume were used (e.g., if the 4 half-life OST value corresponded to the 645^th^ volume and the last acquired volume for that subject was 502, then the beta weights from the 502^th^ volume was used). Each feature had 88 dimensions (22 usable ICs for each of 4 trial types). A comparison set of binary classification features were computed from the GLM-derived beta weights for the full scan duration for each the four trial types.

We implemented leave-one-out cross-validation (LOOCV) in which the training dataset’s labels were balanced. First, we iteratively chose one subject as the testing set in LOOCV. Upon selecting a testing subject, we randomly sampled the CD subjects to match the number of CON subjects. If the testing subject was from the CD group, then 27 out of a possible 40 CD subjects were selected to achieve a balanced training set (n = 27 CD and n = 27 CON, n = 54 total). Similarly, if the testing subject was from CON group, then only 26 out of 40 CD subjects were selected for the cross-validation training set (n = 26 CD and n = 26 CON for n = 52 total). To ensure the random subject selection would not affect the outcome and obtain the estimate of truth accuracy, we sampled and fit 1000 independently drawn training sets per iteration of the LOOCV, averaging the resultant 1000 test predictions to form the test prediction of that LOOCV iteration.

We tested whether the group-wise binary classification accuracies were significantly greater than chance (1-sided, 1-sample Wilcoxon signed-rank test [20]; null = 0.5). We also tested whether classification accuracies trained with OST-related features were significantly greater than those trained on the fully acquired task’s features via pair-wise Wilcoxon signed-rank test [20]. We did not control for gender in our dataset. Hence, to control for gender as a factor, we regressed out participant gender from the vwGLR beta weights and trained the group classifier on the resultant residuals.

### Secondary validation of *t*_1/2_-derived OSTs: behavior correlates of OST-related neural activation patterns

Since the SST is a canonical behavioral measure of motor impulsivity, we tested whether the participants’ SST performances were similarly described by the fully acquired and abbreviated OST-related task lengths. For each trial type (GfSS, GfFS, SS, and FS), we calculated the correlation between participant’s cumulative performance (reaction time, RT) for the full task and task lengths of 1, 2, 3, and 4 half-lives. Note, participants for whom the *t*_1/2_-derived task length included all task data (i.e., the final volume of the *t*_1/2_-estimated task length exceeded the subject’s last acquired volume, as described previously) were excluded from analysis as the resulting perfect correlation between *t*_1/2_-derived, OST-abbreviated tasks and full tasks would unfairly bias the analysis.

## Results

### Inter-individual differences in SST-related beta weight convergence rate

**Fig 4:**
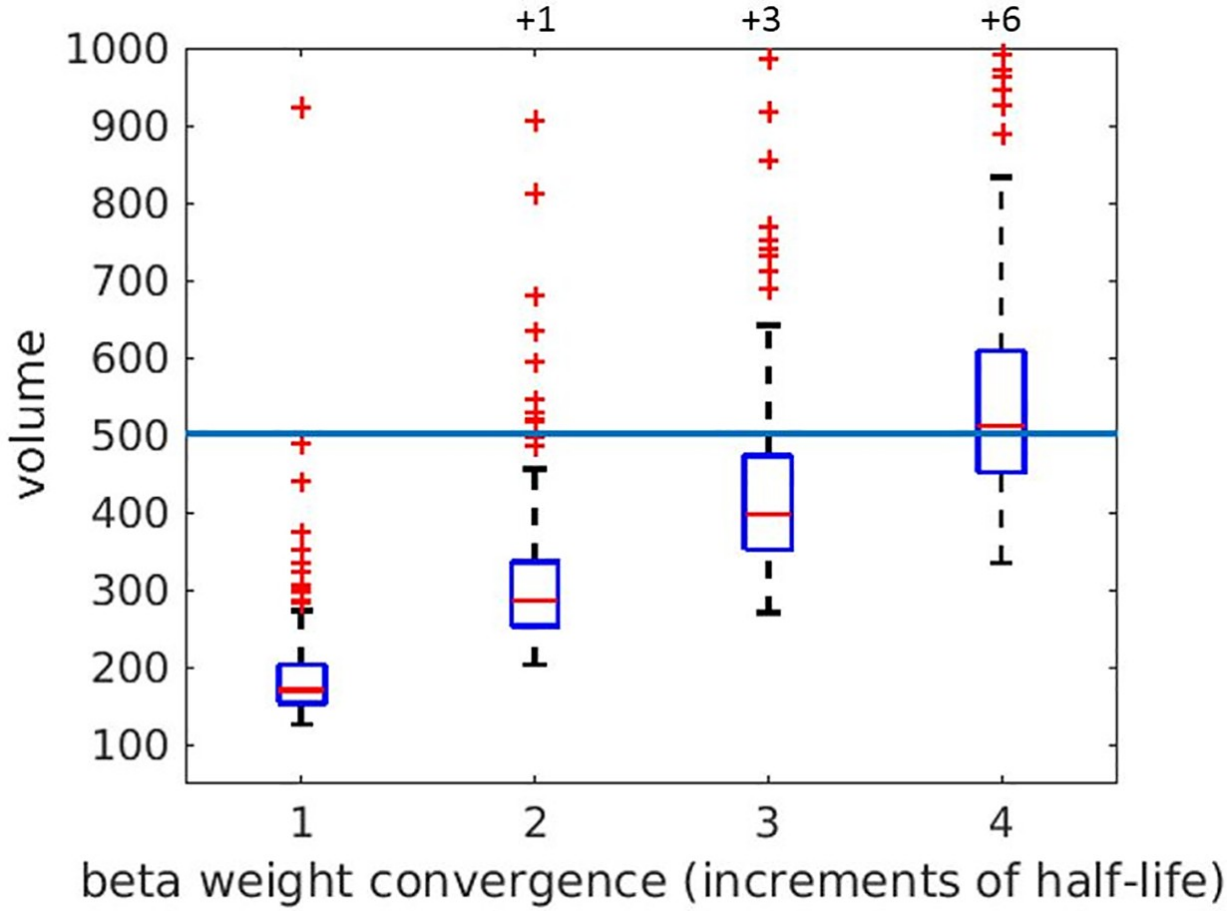
Box plots depict individual OST values for 1, 2, 3, 4 half-lives of SoAD value decay for all 67 subjects. Each box plot depicts the subject means (horizontal line), dynamic range of volume data for the mid 50^th^percentile (box height), range of non-outlier data (vertical bars), and outliers (+s). The full-scale horizontal line indicates the common fully acquired volume model (i.e., 502 TRs); any value above this horizontal line indicates an estimated individual OST that exceeds the limit of the scan session. Values plotted above the figure denote the number of outliers clipped from presentation.

The primary goal of this study was to determine the presence and extent of individual variation in the rate by which iterative presentations of fMRI task trials result in stable functional brain state representations. As a first step, we estimated the individual OST (measured in volumes) for each subject at task lengths of 1, 2, 3 and 4 half-lives. Group-level summaries of these distributions are depicted in Fig 4. Notice modeling 3 and 4 half-lives resulted in many stopping times that exceeded the number of fully acquired volumes; most of the 4 half-life beta weights represent beta weights from the last volume. Therefore, one should expect the OST-trained classification performance from later half-lives to be similar to the classifier performance trained without OSTs (full model). Furthermore, the elongated “boxes” corresponding to 3 and 4 half-lives are due to the fact that 2 or more half-lives are merely multipliers of 1 half-life and hence the distributions of OSTs were widened. Since group comparison outcomes were expected to be similar among various half-lives, we would only use 1 half-life for the following clinical label and trial type effects.

Under a group-wise analysis of 1 half-life convergence rates, the difference between 25^th^ and 75^th^ percentiles of subjects is 49.5 volumes (approximately 99 seconds of scan time). At 2 half-lives, the difference would grow to greater than 3 minutes of scan time, indicating profoundly different beta weight convergence rate between the fastest 25% and the slowest 25% of subjects. Furthermore, there are 12 outliers for which the estimated 1^st^ half-life consumes from 286 volumes to as high as 923 volumes of scan time, corresponding to extremely slow beta weight convergence rates.

**Fig 5:**
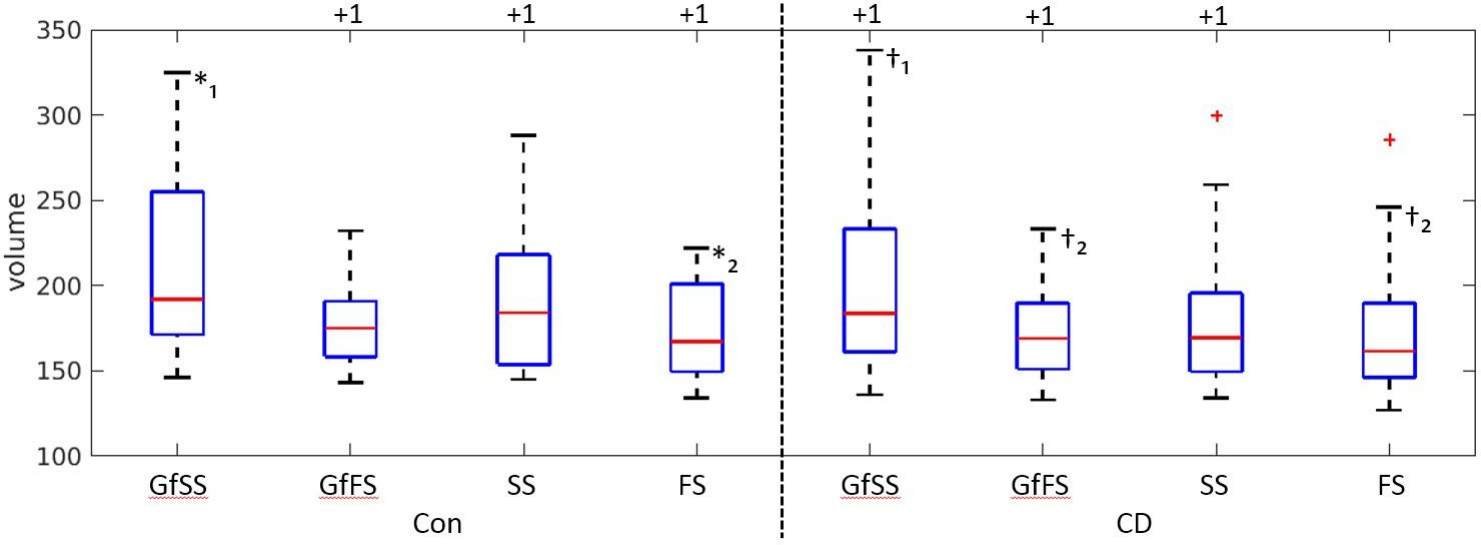
Box plots depict individual OST values computed for 1 half-life of SoAD decay for each clinical label and for each of the four SST trial types. Box plots denote the same analytical content as the analysis of 1 half-life in Fig 4 but divided here by clinical label and trial type. *,†: *₁ is significant from *₂ (Wilcoxon rank-sum test at α = 0.05, p<0.05). Same for †. Values plotted above the figure denote the number of outliers clipped from presentation.

We also examined the role that CD and trial type may play in the rate of beta weight convergence. As shown in Fig 5, CD and CON groups did not exhibit significantly different convergence rates when compared at 1 half-life. However, the distribution of stopping times differed across SST trial types. In the CON group, GfSS trial-related stopping times were significantly greater than FS-related stopping times (Wilcoxon rank-sum test [21], α = 0.05, p<0.05). For the CD group, GfSS trial-related stopping times were significantly greater than both GfFS-related (p<0.05) and FS-related (p<0.01) stopping times.

### Binary classification of CD supports the OST approach for the stop signal task

A second goal of this study was to determine if individual differences in beta-value convergence influence the ability of SST-related neural pattern classification to discriminate individuals with CD. Table 2 depicts mean SVM-derived neural pattern classification accuracy for each trial type and each task length (estimated in either unit of half-life or full task duration). Note that these accuracies were averaged over LOOCV iterations (67 total classifications). Among the four tested half-lives, modeling OST values corresponding to 2 half-lives produced the highest accuracy where all four trial types were associated with average CD vs CON classification accuracies that were significantly greater than chance (one-sided Wilcoxon signed-rank test [20] at α = 0.05, p<0.05). As expected, we obtained similar classifier performance for task lengths based on 3 and 4 half-lives in comparison to the fully acquired data set, since they were largely trained and tested using the full task duration beta weights (see Fig 4). Here, none of the trial type and half-life combinations produced significantly greater or poorer binary classification performance than the full task (pairwise Wilcoxon signed-rank test [20] at α = 0.05, Bonferroni corrected).

**Table 2:** The average CD vs CON LOOCV neural pattern classification accuracies of *t*_1/2_-derived and Full Task related neural activation patterns conditioned on the determinant of task duration.

		trial type		
half-life	GfSS	GfFS	SS	FS
1	0.577	0.5513	0.5836	0.5686
2	0.6049^a^	0.6087^a^	0.6109^a^	0.6611^a^
3	0.5551	0.5866^a^	0.606^a^	0.5812^a^
4	0.5692	0.5862	0.5619	0.6041^a^
full task		0.5723		

^a^: indicating the average classification accuracy values from LOOCV was significantly greater than chance (one-sided Wilcoxon signed-rank test, p<0.05, null = 0.5).

When controlling for sex differences (Table 3), the OST values defined by 2 half-lives again produced overall the best classification accuracy, with three of the four trial types yielding average CD classification accuracies that were significantly greater (one-sided Wilcoxon signed-rank test [20] at α = 0.05, p<0.05) than chance; moreover, the classification features built from beta-values estimated at the 2 half-life task length determined for GfSS and GfFS trial types produced average accuracies that were significantly greater than classifiers trained on the fully acquired trials (pairwise Wilcoxon signed-rank test [20] at α = 0.05, p<0.05, Bonferroni corrected).

**Table 3:** The average CD vs CON LOOCV classification accuracies of OST-derived and Full Task derived neural activation patterns after regressing out the effect of participant sex from the beta weights.

	trial type			
half-life	GfSS	GfFS	SS	FS
1	0.5592	0.5200	0.5576	0.5220
2	0.6694 ^a^^b^	0.7133^a^^b^	0.5619	0.5837^a^
3	0.5371	0.5360	0.5668	0.5482
4	0.5425	0.5416	0.5456	0.5626
full task	0.5450			

^a^: indicating the average classification accuracy values from LOOCV was significantly greater than chance (one-sided Wilcoxon signed-rank test, p<0.05, null = 0.5).

^b^: indicating those *t*_1/2_-derived classification accuracies from LOOCV were significantly greater than accuracies obtained from the fully acquired model (pair-wise Wilcoxon signed-rank test, p<0.05, Bonferroni corrected).

### Behavioral outcome

The participants’ SST behavioral performance measures for the full task trials and *t*_1/2_-abbreviated task trials (Table 4) remained highly correlated across all task conditions and half-lives (all r≥0.94, all p≤0.001). Note, no results are reported for SS since the SS trial type does not yield reaction time values.

**Table 4:** Pearson correlation coefficients between participant’s reaction time values for the full task and each *t*_1/2_-derived task for each trial type.

	trial type		
half-life	GfSS	GfFS	FS
1	0.9441	0.9554	0.9433
2	0.9787	0.9705	0.9699
3	0.9864	0.9895	0.9892
4	0.9898	0.9978	0.9962

All of correlation of reaction time between full task and *t*_1/2_-derived task are significantly correlated (all r≥0.94, all p≤0.001).

## Discussion

With the introduction of fMRI designs involving highly sampled individual subjects has come a profound appreciation of the remarkable individuality of functional organization of the human brain [5,22,23]. Driven largely on the basis of resting state fMRI data, this individuation motif has now been demonstrated for task-based fMRI studies as well [24]. In this study, we sought to explore possible individual differences in task-related fMRI responses to a commonly applied fMRI task, the Stop Signal Task (SST), but at the level of the temporal development of stable neural responses to task-related processing demands. This individual brain-behavior response variable has been proposed in theory [3] and simulation [25] but has to our knowledge not been deployed in practice, either prospectively nor retrospectively.

The great majority of task activation fMRI studies have centered on defining functional neuroanatomy for the group-average brain using task paradigms with pre-specified numbers of trials, stimulus presentations, and task durations. This study addresses whether the assumptions underlying pre-specification hold. Specifically, we hypothesized that the brain response dynamics to stimulus presentations differ across individuals. To address this potentially important source of individual differences in task-related fMRI, we sought to simulate a real-time measurement of trial-by-trial neural response via volume-by-volume analysis of a previously acquired SST data set. The outcome variable of interest was an individual OST for four discrete SST trial types corresponding to the functions of response inhibition, errors of commission, and next-trial responses to both outcomes [26].

The volume-by-volume analysis of SST fMRI data supported the presence of individual differences in the acquisition rate of stable task-related neural responses. As such, some subjects may readily acquire the neural processing representation of the task demand and subsequently respond to stimuli with reduced attention or shift to brain mechanisms associated with an automaticity of response organization. Conversely, other subjects fail to fully obtain a stable neural processing representation for the pre-defined task trials, adding another level of variance to the group-averaged data. Pooling such diversity of stimulus responses in post-hoc analysis may blur the target brain-behavior relationship. We found some support for this concept of diminishing returns with stimulus presentations. Many of the canonical SST durations continued beyond our best estimated OST. Moreover, neural pattern classification of CD accuracy was maximized when conducted on OST-abbreviated training data for several SST trial types relative to classifiers trained on the fully acquired data.

Indeed, we report a wide range in estimated converge rates, with some individual OST estimates exceeding the total number of acquired volumes (i.e., 502) before the first half-life (i.e. 50% of the total expected change of neural activation) of brain state convergence. The existence of this extremely slow beta weight convergence rate (e.g., 1 half-life in 923 volumes) demonstrates that some subjects require either extended task durations to achieve stable neural activation pattern, reassessment of task comprehension and restart, or even omission of their data from the study analysis due to task engagement failure. In addition to marked individual differences in estimated convergence rate, we also observed that each SST trial type exhibited unique rates of convergence, indicating that different SST processes are associated with differing temporal aspects of neural information processing. This observation suggests that fMRI designs associated with distinct task-based processing demands do not equivalently characterize their neural representations within fixed stimulus paradigms.

Our individual differences approach to fMRI acquisition is similar to that of a recent study [4], which sought to develop an individualized delay discounting task (IDT) for the fMRI environment that reduced in-scanner time without compromising the fidelity of its underlying neural correlates. By modeling the indifference curves related to the relative value of immediate and delayed rewards with an ‘S’ shaped hyperbolic function, the IDT would stop acquiring images once it had sufficient behavioral data to estimate the function’s parameters. This and the current approach are similar in several ways: 1) In a trial-by-trial manner, we also sought to define the individual optimal stopping time for image acquisitions without compromising statistical power to define the neural response; and 2) we also utilized mathematical functions to model the “individuality” of the neural responses among subjects. However, while the stopping point for image acquisition for the IDT task is based on attaining a behavioral endpoint, the current study focused on attaining a neural activation pattern as the stopping endpoint. Thus, while the IDT focused specifically on the delay discounting task, our approach is designed to be broadly applicable to any task using general linear regression to model fMRI task-related neural activation. Furthermore, the current work explored both inter-subject differences and intra-subject inter-trial differences for a deeper understanding of the potential costs and benefits of this approach to individualizing image acquisition parameters.

We sought to address the question of whether the decrease in number of modeled events associated with using OST-abbreviated image volume acquisitions sacrifices the fMRI information content related to defining the neural processing correlates of target behaviors. Specifically, we assessed whether individually abbreviated stimulus presentations defined comparable brain-behavior relationships relative to conventional fixed stimulus regimens by comparing the accuracy of neural pattern classification of cocaine dependence between *t*_1/2_-abbreviated and fully acquired fMRI data for each of the four SST trial types. For individual *t*_1/2_-derived neural activations at two half-lives of exponential decay of SoAD values, binary classification accuracy of CD individuals from healthy controls for each of the four SST trial types significantly exceeded chance. Average classification accuracy was comparable to that obtained for classifiers trained on the full fMRI data set, and significantly more accurate for GfSS and GfFS trials if sex differences are regressed out. When fixing the OST at 2 half-lives, individual OST-abbreviated image volumes have at least equivalent neural processing information content to that of the larger, fully acquired imaging datasets. Furthermore, SST reaction time data were highly correlated between the full and OST-truncated tasks, supporting that informed task truncation does not impact the rich behavioral output associated with this task.

It is noteworthy that the average classification accuracies for DSM-IV CD in the present study using balanced groups were lower than those obtained using a separate approach to multivariate pattern analysis (linear discriminant function analysis) with unbalanced, male-only CD and HC groups [7]. There are several possible explanations for the lower than expected classification accuracies reported in Tables 2 and 3 (ranged from 52% to 71%). The current study chose the least statistically biased validation method—leave one out cross-validation. Also, compared to our previous work [7], which utilized an unbalanced training set, we utilized a balanced training set in generating the binary classifier, which generally produces less biased classifiers [27,28].

Defining the individualized optimal stopping point for stimulus presentations would reduce in-scanner time and thus potentially enhance the acceptability and tolerability of task-related fMRI study protocols. Capturing individual differences in the rate of acquiring stable functional brain responses would yield a better standardized neural processing correlate and perhaps improve data quality by minimizing the contribution of other neural processing events related to continued stimulus presentations after the target neural response is acquired. Capturing this source of individual variation represents an as-yet un-tapped solution to the “power problem” attributed to fMRI studies [29].

This fMRI study of OST holds significant consequences for the wider deployment of real-time methodology for task-based fMRI; however, as our conclusions are drawn from retrospective analysis, caution is warranted in directly implementing these findings. Hence, a secondary analysis of existing fMRI signal does not represent a definitive test of the potential costs and benefits associated with actual implementation of an OST approach in real-time, though the study findings motivate such an application. A clear next step is to implement our OST protocol in a dynamic format within a real-time fMRI context. A perhaps more practical application of the retrospective approach to OST estimation used here would its implementation in image pre-processing pipelines as a means of controlling for the noise associated with individual and group-level rates in acquiring stable neural processing representations of tasks demands.

## Conclusions

The findings of this study indicate that individual differences in the temporal rate of acquiring stable neural activation patterns representing the processing demands posed by tasks in fMRI studies represent a typically hidden variable that affects study tolerability, outcomes, and inferences, and adds to the argument for individual-level approaches to characterizing human functional brain organization [22,24]. The study findings also question the value of the common approach of employing pre-specified numbers of task trials in fMRI study designs.

## Supporting information

S1 Fig A comparison of outcomes from ordinary (LR) and robust regression (robust LR). (TIF)
