# Supplementary figures and images for: Individual differences in rate of acquiring stable neural representations of tasks in fMRI
Source: PLoS One. 2018 Nov 26;13(11):e0207352. doi: 10.1371/journal.pone.0207352 (PMC6261022; doi:10.1371/journal.pone.0207352)

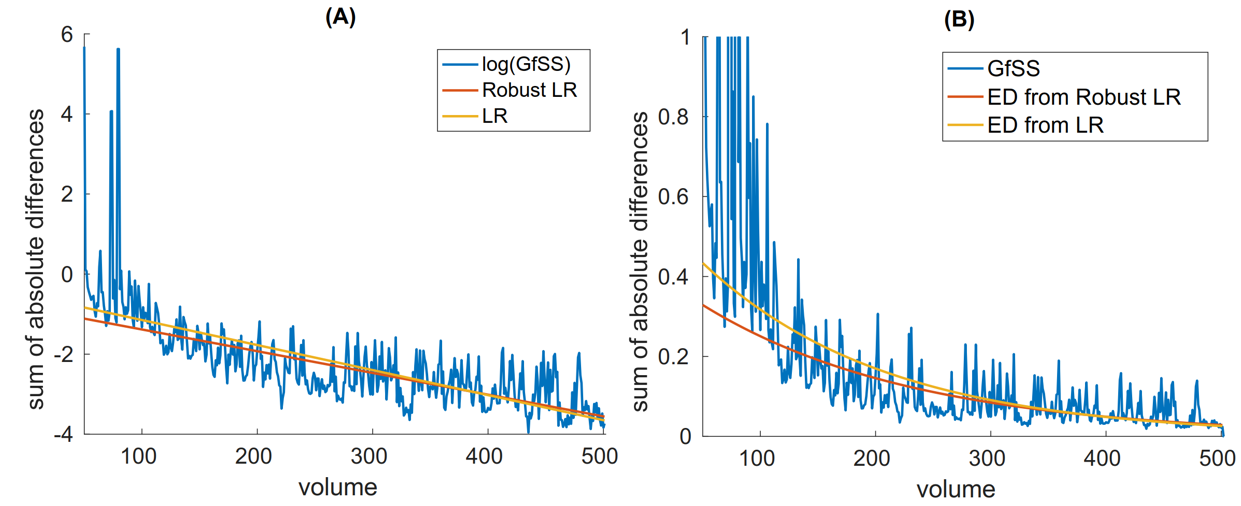

Supplement: S1 Fig — (TIF) [file pone.0207352.s001.tif]
